# Reconstruction of cellular variability from spatiotemporal patterns of *Dictyostelium discoideum*

**DOI:** 10.1186/1753-4631-1-10

**Published:** 2007-08-30

**Authors:** Christiane Hilgardt, Stefan C Müller, Marc-Thorsten Hütt

**Affiliations:** 1 Biophysics Group, Institute of Experimental Physics, Otto-von-Guericke University, Magdeburg, Germany; 2 School of Engineering and Science, Jacobs University Bremen, Germany

## Abstract

Variability in cell properties can be an important driving mechanism behind spatiotemporal patterns in biological systems, as the degree of cell-to-cell differences determines the capacity of cells to locally synchronize and, consequently, form patterns on a larger spatial scale. In principle, certain features of spatial patterns emerging with time may be regulated by variability or, more specifically, by certain constellations of cell-to-cell differences. Similarly, measuring variability in a system (i.e. the spatial distribution of cell-cell differences) may help predict properties of later-stage patterns.

Here we apply and compare different statistical methods of extracting such systematic cell-to-cell differences in the case of patterns generated with a simple model system of an excitable medium and of experimental data by the slime mold *Dictyostelium discoideum*. We demonstrate with the help of a correlation analysis that these methods produce systematic (i.e. stationary) results for cell properties. Furthermore, we discuss possible applications of our method, in particular how these cell properties may serve as predictors of certain later-stage patterns.

## Background

In biological pattern formation a process of self-organization and a breaking of spatial symmetry are sometimes related. In physics symmetry breaking is often triggered by random fluctuations, enabling the system to select a particular stable steady state. In biology, however, differences between the constituents of the system may in a sense pre-determine the outcome of symmetry breaking and can in principle allow predicting the layout of resulting patterns. This possibility to translate cellular variability into features of patterns requires new methods of analyzing spatiotemporal data. One set of methods in the core of this endeavor, the reconstruction of variability distributions from data, is the topic of the present paper.

In theoretical studies variability (sometimes refered to as disorder) is now appreciated as a source of randomness that, similarly to noise, can interact with the non-linearities of the system and systematically influence patterns. For noise such influences are well known: Noise-induced transitions and even noise-enhanced structure formation have been explored in theoretical model systems [1,2] as well as in nature (see, e.g., [3] for an overview of activities in this field related to biology). Remarkably, this discussion of stochastic contributions acting constructively can be found on all spatial and temporal scales, from the genetic level to the molecular and cellular levels and up to the level of ecological pattern formation. If we discuss a multicellular system, variability can be thought of as the spatial distribution of systematic cell-to-cell differences. In contrast to dynamic noise, biological variability is a static system property. In order to study the effect of variability on patterns in a biological system, one has to reconstruct variability distributions from observable spatiotemporal data. For better understanding the details involved in reconstruction, it is convenient to complement the design of new analysis techniques by sample data generated by mathematical model systems and then putting similar restrictions on these sample data as in the case of an actual experiment. With the help of such sample data one can test, how well the analysis tools are capable of handling experimental data [4,5]. A model system, for which a regulation by variability could be a principal mechanism (see, e.g., [6,7]), is the slime mold *Dictyostelium discoideum*. In this example of biological pattern formation individual cells aggregate under the influence of the chemotactic signal cAMP and form a multicellular organism [8]. Under nutrient deprivation single cells start to emit cAMP into the environment. The molecules are detected by neighboring cells via highly specific surface receptors [9]. This initiates the intracellular autocatalytic synthesis of additional cAMP by the enzyme adenylat cyclase (ACA) and the subsequent segregation into the environment. Time delayed receptor desensitization and stopping of ACA activity are involved in the following refractory period. Extracellular cAMP is degraded by membrane-bound and segregated phosphodiesterase, which is on the other hand regulated by its inhibitor. The coupling of the underlying reaction kinetics with diffusion results in wave propagation. As long as the local cAMP concentration increases in time, the cells react with positive chemotaxis, resulting in the periodic movement perpendicular to the wave front, i.e. towards the origin of the chemical signal. The process of aggregation is accompanied by target patterns and spiral waves of cAMP [10,11] and, at a later stage, cell streams leading from the periphery to the center of the territory [12-14]. At a later stage the cells have accumulated to a cell mound in the center of the aggregation territory. The advanced stages of the developmental cycle include transitions into successive types of multicellular aggregates and finally the formation of a differentiated fruiting body with spores capable of germination (see [15] for detailed information on *D. discoideum *life cycle).

We believe that in the case of *D. discoideum *variability is responsible for certain stages of symmetry breaking in the usual course of the developmental cycle (local pattern initiation, spatial distribution of cell streams, distribution and proportions of differentiated cell types). A strong support for our hypothesis that indeed cell properties can affect the collective patterns in *D. discoideum *has come from the recent observation that direction and magnitude of a cell's response to a signal pulse is indeed a cell property, which remains constant in time [16] and therefore falls into the general scheme outlined within the present paper. In that work the behavior of single cells under periodic cAMP signals has been analyzed and it is observed that the characteristics of the gradient sensing response of an individual cell at a certain time point strongly correlates with that of the same cell at a later time point.

The structure of our paper is as follows: First we will formulate spatiotemporal analysis tools capable of extracting distributions of cellular properties (Section 2). Next we will study for a simple model system of excitable media (Section 3) how well pre-defined distributions can be reconstructed from the simulated spatiotemporal patterns with the help of these analysis tools (Section 5.1) and lastly we will apply these tools to measured wave patterns for *D. discoideum *(Section 4) and show that the reconstructed distributions are indeed systematic: distributions found for a particular time window strongly correlate with those distributions of the same observable obtained for other time windows (Section 5.2). In the conclusions (Section 6) we place the process of variability reconstruction into the larger context of prediction schemes.

## Spatiotemporal analysis tools

### CA fluctuation number

Cellular automata (CA) are a useful mathematical approach for studying, by numerical simulation, the global patterns arising in a system on the basis of certain local interactions [17,18]. In a series of previous studies spatiotemporal filters have been formulated translating CA-type neighborhood constellations into quantitative estimates of a certain system property [19]. We have used these tools to study the phenomenon of spatiotemporal stochastic resonance [20], to quantify synchronization properties of biological patterns [21] and to develop an algorithm for evaluating independently the contributions of measurement noise and internal noise to a spatiotemporal data set [22]. All these applications were based upon the temporal change of space-averaged observables.

Here we will formulate and study a spatially explicit variant of this fluctuation analysis and apply it to patterns from excitable media. These tools can form a basis for identifying and quantifying variability in spatiotemporal data sets from biological systems.

Let ℐ
 MathType@MTEF@5@5@+=feaafiart1ev1aaatCvAUfKttLearuWrP9MDH5MBPbIqV92AaeXatLxBI9gBaebbnrfifHhDYfgasaacH8akY=wiFfYdH8Gipec8Eeeu0xXdbba9frFj0=OqFfea0dXdd9vqai=hGuQ8kuc9pgc9s8qqaq=dirpe0xb9q8qiLsFr0=vr0=vr0dc8meaabaqaciaacaGaaeqabaqabeGadaaakeaat0uy0HwzTfgDPnwy1egaryqtHrhAL1wy0L2yHvdaiqaacqWFqessaaa@3768@ denote a two-dimensional spatial data set, i.e. a square matrix of size *N *with components *a*_*ij *_∈ Σ, where Σ is the set of possible states. The restriction to a square matrix is only imposed for notational convenience. A time sequence of such matrices (or "images") is a set {ℐ
 MathType@MTEF@5@5@+=feaafiart1ev1aaatCvAUfKttLearuWrP9MDH5MBPbIqV92AaeXatLxBI9gBaebbnrfifHhDYfgasaacH8akY=wiFfYdH8Gipec8Eeeu0xXdbba9frFj0=OqFfea0dXdd9vqai=hGuQ8kuc9pgc9s8qqaq=dirpe0xb9q8qiLsFr0=vr0=vr0dc8meaabaqaciaacaGaaeqabaqabeGadaaakeaat0uy0HwzTfgDPnwy1egaryqtHrhAL1wy0L2yHvdaiqaacqWFqessaaa@3768@(*t*) ; *t *= 1, 2, ..., *N*_*T*_}, again with some normalized (dimensionless) time *t *and the number of images *N*_*T *_in the sequence. We formulate a spatiotemporal filter, which approximates the contribution of noise to an observed dynamics by studying the relative movement of neighbors of a particular cell aij(t)
 MathType@MTEF@5@5@+=feaafiart1ev1aaatCvAUfKttLearuWrP9MDH5MBPbIqV92AaeXatLxBI9gBaebbnrfifHhDYfgasaacH8akY=wiFfYdH8Gipec8Eeeu0xXdbba9frFj0=OqFfea0dXdd9vqai=hGuQ8kuc9pgc9s8qqaq=dirpe0xb9q8qiLsFr0=vr0=vr0dc8meaabaqaciaacaGaaeqabaqabeGadaaakeaacqWGHbqydaqhaaWcbaGaemyAaKMaemOAaOgabaGaeiikaGIaemiDaqNaeiykaKcaaaaa@33FF@ at a time *t*, i.e. by looking at changes of the quantities δij(t,k)
 MathType@MTEF@5@5@+=feaafiart1ev1aaatCvAUfKttLearuWrP9MDH5MBPbIqV92AaeXatLxBI9gBaebbnrfifHhDYfgasaacH8akY=wiFfYdH8Gipec8Eeeu0xXdbba9frFj0=OqFfea0dXdd9vqai=hGuQ8kuc9pgc9s8qqaq=dirpe0xb9q8qiLsFr0=vr0=vr0dc8meaabaqaciaacaGaaeqabaqabeGadaaakeaaiiGacqWF0oazdaqhaaWcbaGaemyAaKMaemOAaOgabaGaeiikaGIaemiDaqNaeiilaWIaem4AaSMaeiykaKcaaaaa@369F@ in

δij(t)={aij(t)−b(t); b(t)∈Nij}={δij(t,1),...,δij(t,|Nij|)},
 MathType@MTEF@5@5@+=feaafiart1ev1aaatCvAUfKttLearuWrP9MDH5MBPbIqV92AaeXatLxBI9gBamXvP5wqSXMqHnxAJn0BKvguHDwzZbqegyvzYrwyUfgarqqtubsr4rNCHbGeaGqiA8vkIkVAFgIELiFeLkFeLk=iY=Hhbbf9v8qqaqFr0xc9pk0xbba9q8WqFfeaY=biLkVcLq=JHqVepeea0=as0db9vqpepesP0xe9Fve9Fve9GapdbaqaaeGacaGaaiaabeqaamqadiabaaGcbaacciGae8hTdq2aa0baaSqaaiabdMgaPjabdQgaQbqaaiabcIcaOiabdsha0jabcMcaPaaakiabg2da9iabcUha7jabdggaHnaaDaaaleaacqWGPbqAcqWGQbGAaeaacqGGOaakcqWG0baDcqGGPaqkaaGccqGHsislcqWGIbGydaahaaWcbeqaaiabcIcaOiabdsha0jabcMcaPaaakiabcUda7iabbccaGiabdkgaInaaCaaaleqabaGaeiikaGIaemiDaqNaeiykaKcaaOGaeyicI48enfgDOvwBHrxAJfwnHbqeh0uy0HwzTfgDPnwy1aacfaGae4xdX70aaSbaaSqaaiabdMgaPjabdQgaQbqabaGccqGG9bqFcqGH9aqpcqGG7bWEcqWF0oazdaqhaaWcbaGaemyAaKMaemOAaOgabaGaeiikaGIaemiDaqNaeiilaWIaeGymaeJaeiykaKcaaOGaeiilaWIaeiOla4IaeiOla4IaeiOla4IaeiilaWIae8hTdq2aa0baaSqaaiabdMgaPjabdQgaQbqaaiabcIcaOiabdsha0jabcYcaSiabcYha8jab+1q8onaaBaaameaacqWGPbqAcqWGQbGAaeqaaSGaeiiFaWNaeiykaKcaaOGaeiyFa0NaeiilaWcaaa@8F33@

which serves as a means of separating directed and undirected (eventually stochastic) change of the state of a cell. If the discretization of the spatiotemporal data set in space (due to the finite cell size) and time (due to the finite number of images) is small enough, directed and stochastic changes will have very different scales in time and space. For means of separation, one has to assume that the scales for the stochastic part will be smaller than the scales present in the discretization of the data set and the time scales of deterministic dynamics themselves. This leads to a (suffcient) condition for a manifestation of noise in a specific change at aij(t)
 MathType@MTEF@5@5@+=feaafiart1ev1aaatCvAUfKttLearuWrP9MDH5MBPbIqV92AaeXatLxBI9gBaebbnrfifHhDYfgasaacH8akY=wiFfYdH8Gipec8Eeeu0xXdbba9frFj0=OqFfea0dXdd9vqai=hGuQ8kuc9pgc9s8qqaq=dirpe0xb9q8qiLsFr0=vr0=vr0dc8meaabaqaciaacaGaaeqabaqabeGadaaakeaacqWGHbqydaqhaaWcbaGaemyAaKMaemOAaOgabaGaeiikaGIaemiDaqNaeiykaKcaaaaa@33FF@

Sig[δij(t,k)−δij(t−1,k)]≠Sig[δij(t+1,k)−δij(t,k)]∧δij(t,k)−δij(t−1,k)≠0∧δij(t+1,k)−δij(t,k)≠0,
 MathType@MTEF@5@5@+=feaafiart1ev1aaatCvAUfKttLearuWrP9MDH5MBPbIqV92AaeXatLxBI9gBaebbnrfifHhDYfgasaacH8akY=wiFfYdH8Gipec8Eeeu0xXdbba9frFj0=OqFfea0dXdd9vqai=hGuQ8kuc9pgc9s8qqaq=dirpe0xb9q8qiLsFr0=vr0=vr0dc8meaabaqaciaacaGaaeqabaqabeGadaaakeaacqqGtbWucqqGPbqAcqqGNbWzdaWadaqaaGGaciab=r7aKnaaDaaaleaacqWGPbqAcqWGQbGAaeaacqGGOaakcqWG0baDcqGGSaalcqWGRbWAcqGGPaqkaaGccqGHsislcqWF0oazdaqhaaWcbaGaemyAaKMaemOAaOgabaGaeiikaGIaemiDaqNaeyOeI0IaeGymaeJaeiilaWIaem4AaSMaeiykaKcaaaGccaGLBbGaayzxaaGaeyiyIKRaee4uamLaeeyAaKMaee4zaC2aamWaaeaacqWF0oazdaqhaaWcbaGaemyAaKMaemOAaOgabaGaeiikaGIaemiDaqNaey4kaSIaeGymaeJaeiilaWIaem4AaSMaeiykaKcaaOGaeyOeI0Iae8hTdq2aa0baaSqaaiabdMgaPjabdQgaQbqaaiabcIcaOiabdsha0jabcYcaSiabdUgaRjabcMcaPaaaaOGaay5waiaaw2faaiabgEIizlab=r7aKnaaDaaaleaacqWGPbqAcqWGQbGAaeaacqGGOaakcqWG0baDcqGGSaalcqWGRbWAcqGGPaqkaaGccqGHsislcqWF0oazdaqhaaWcbaGaemyAaKMaemOAaOgabaGaeiikaGIaemiDaqNaeyOeI0IaeGymaeJaeiilaWIaem4AaSMaeiykaKcaaOGaeyiyIKRaeGimaaJaey4jIKTae8hTdq2aa0baaSqaaiabdMgaPjabdQgaQbqaaiabcIcaOiabdsha0jabgUcaRiabigdaXiabcYcaSiabdUgaRjabcMcaPaaakiabgkHiTiab=r7aKnaaDaaaleaacqWGPbqAcqWGQbGAaeaacqGGOaakcqWG0baDcqGGSaalcqWGRbWAcqGGPaqkaaGccqGHGjsUcqaIWaamcqGGSaalaaa@9E59@

where the last two inequalities are subsidiary conditions introduced for convenience and the sign function Sig [*x*] gives 1, -1 or 0, if *x *is larger than, less than or equal to zero, respectively. Each transition δij(t−1,k)→δij(t,k)→δij(t+1,k)
 MathType@MTEF@5@5@+=feaafiart1ev1aaatCvAUfKttLearuWrP9MDH5MBPbIqV92AaeXatLxBI9gBaebbnrfifHhDYfgasaacH8akY=wiFfYdH8Gipec8Eeeu0xXdbba9frFj0=OqFfea0dXdd9vqai=hGuQ8kuc9pgc9s8qqaq=dirpe0xb9q8qiLsFr0=vr0=vr0dc8meaabaqaciaacaGaaeqabaqabeGadaaakeaaiiGacqWF0oazdaqhaaWcbaGaemyAaKMaemOAaOgabaGaeiikaGIaemiDaqNaeyOeI0IaeGymaeJaeiilaWIaem4AaSMaeiykaKcaaOGaeyOKH4Qae8hTdq2aa0baaSqaaiabdMgaPjabdQgaQbqaaiabcIcaOiabdsha0jabcYcaSiabdUgaRjabcMcaPaaakiabgkziUkab=r7aKnaaDaaaleaacqWGPbqAcqWGQbGAaeaacqGGOaakcqWG0baDcqGHRaWkcqaIXaqmcqGGSaalcqWGRbWAcqGGPaqkaaaaaa@520A@ fulfilling the condition (2) gives a contribution

12(|δij(t,k)−δij(t−1,k)|+|δij(t+1,k)−δij(t,k)|).
 MathType@MTEF@5@5@+=feaafiart1ev1aaatCvAUfKttLearuWrP9MDH5MBPbIqV92AaeXatLxBI9gBaebbnrfifHhDYfgasaacH8akY=wiFfYdH8Gipec8Eeeu0xXdbba9frFj0=OqFfea0dXdd9vqai=hGuQ8kuc9pgc9s8qqaq=dirpe0xb9q8qiLsFr0=vr0=vr0dc8meaabaqaciaacaGaaeqabaqabeGadaaakeaadaWcaaqaaiabigdaXaqaaiabikdaYaaadaqadaqaamaaemaabaacciGae8hTdq2aa0baaSqaaiabdMgaPjabdQgaQbqaaiabcIcaOiabdsha0jabcYcaSiabdUgaRjabcMcaPaaakiabgkHiTiab=r7aKnaaDaaaleaacqWGPbqAcqWGQbGAaeaacqGGOaakcqWG0baDcqGHsislcqaIXaqmcqGGSaalcqWGRbWAcqGGPaqkaaaakiaawEa7caGLiWoacqGHRaWkdaabdaqaaiab=r7aKnaaDaaaleaacqWGPbqAcqWGQbGAaeaacqGGOaakcqWG0baDcqGHRaWkcqaIXaqmcqGGSaalcqWGRbWAcqGGPaqkaaGccqGHsislcqWF0oazdaqhaaWcbaGaemyAaKMaemOAaOgabaGaeiikaGIaemiDaqNaeiilaWIaem4AaSMaeiykaKcaaaGccaGLhWUaayjcSdaacaGLOaGaayzkaaGaeiOla4caaa@658A@

Averaging with respect to *k *leads to the final expression for the spatially explicit version Ω_*ij*_*(t*) of the CA fluctuation number Ω(*t*):

Ωij(t)=1|Nij|∑k=1|Nij|12(|δij(t,k)−δij(t−1,k)|+|δij(t+1,k)−δij(t,k)|)×12Sig[δij(t,k)−δij(t−1,k)]Sig[δij(t+1,k)−δij(t,k)](Sig[δij(t,k)−δij(t−1,k)]Sig[δij(t+1,k)−δij(t,k)]−1),
 MathType@MTEF@5@5@+=feaafiart1ev1aaatCvAUfKttLearuWrP9MDH5MBPbIqV92AaeXatLxBI9gBaebbnrfifHhDYfgasaacH8akY=wiFfYdH8Gipec8Eeeu0xXdbba9frFj0=OqFfea0dXdd9vqai=hGuQ8kuc9pgc9s8qqaq=dirpe0xb9q8qiLsFr0=vr0=vr0dc8meaabaqaciaacaGaaeqabaqabeGadaaakeaafaqaaeGabaaabaGaeuyQdC1aaSbaaSqaaiabdMgaPjabdQgaQbqabaGccqGGOaakcqWG0baDcqGGPaqkcqGH9aqpdaWcaaqaaiabigdaXaqaamaaemaabaWenfgDOvwBHrxAJfwnHbqeg0uy0HwzTfgDPnwy1aaceaGae8xdX70aaSbaaSqaaiabdMgaPjabdQgaQbqabaaakiaawEa7caGLiWoaaaWaaabCaeaadaWcaaqaaiabigdaXaqaaiabikdaYaaaaSqaaiabdUgaRjabg2da9iabigdaXaqaamaaemaabaGae8xdX70aaSbaaWqaaiabdMgaPjabdQgaQbqabaaaliaawEa7caGLiWoaa0GaeyyeIuoakmaabmaabaWaaqWaaeaaiiGacqGF0oazdaqhaaWcbaGaemyAaKMaemOAaOgabaGaeiikaGIaemiDaqNaeiilaWIaem4AaSMaeiykaKcaaOGaeyOeI0Iae4hTdq2aa0baaSqaaiabdMgaPjabdQgaQbqaaiabcIcaOiabdsha0jabgkHiTiabigdaXiabcYcaSiabdUgaRjabcMcaPaaaaOGaay5bSlaawIa7aiabgUcaRmaaemaabaGae4hTdq2aa0baaSqaaiabdMgaPjabdQgaQbqaaiabcIcaOiabdsha0jabgUcaRiabigdaXiabcYcaSiabdUgaRjabcMcaPaaakiabgkHiTiab+r7aKnaaDaaaleaacqWGPbqAcqWGQbGAaeaacqGGOaakcqWG0baDcqGGSaalcqWGRbWAcqGGPaqkaaaakiaawEa7caGLiWoaaiaawIcacaGLPaaacqGHxdaTaeaadaWcaaqaaiabigdaXaqaaiabikdaYaaacqqGtbWucqqGPbqAcqqGNbWzdaWadaqaaiab+r7aKnaaDaaaleaacqWGPbqAcqWGQbGAaeaacqGGOaakcqWG0baDcqGGSaalcqWGRbWAcqGGPaqkaaGccqGHsislcqGF0oazdaqhaaWcbaGaemyAaKMaemOAaOgabaGaeiikaGIaemiDaqNaeyOeI0IaeGymaeJaeiilaWIaem4AaSMaeiykaKcaaaGccaGLBbGaayzxaaGaee4uamLaeeyAaKMaee4zaC2aamWaaeaacqGF0oazdaqhaaWcbaGaemyAaKMaemOAaOgabaGaeiikaGIaemiDaqNaey4kaSIaeGymaeJaeiilaWIaem4AaSMaeiykaKcaaOGaeyOeI0Iae4hTdq2aa0baaSqaaiabdMgaPjabdQgaQbqaaiabcIcaOiabdsha0jabcYcaSiabdUgaRjabcMcaPaaaaOGaay5waiaaw2faamaabmaabaGaee4uamLaeeyAaKMaee4zaC2aamWaaeaacqGF0oazdaqhaaWcbaGaemyAaKMaemOAaOgabaGaeiikaGIaemiDaqNaeiilaWIaem4AaSMaeiykaKcaaOGaeyOeI0Iae4hTdq2aa0baaSqaaiabdMgaPjabdQgaQbqaaiabcIcaOiabdsha0jabgkHiTiabigdaXiabcYcaSiabdUgaRjabcMcaPaaaaOGaay5waiaaw2faaiabbofatjabbMgaPjabbEgaNnaadmaabaGae4hTdq2aa0baaSqaaiabdMgaPjabdQgaQbqaaiabcIcaOiabdsha0jabgUcaRiabigdaXiabcYcaSiabdUgaRjabcMcaPaaakiabgkHiTiab+r7aKnaaDaaaleaacqWGPbqAcqWGQbGAaeaacqGGOaakcqWG0baDcqGGSaalcqWGRbWAcqGGPaqkaaaakiaawUfacaGLDbaacqGHsislcqaIXaqmaiaawIcacaGLPaaacqGGSaalaaaaaa@078A@

where the term in the second row is either 0 or 1 filtering the dynamics according to the fluctuation condition (2). This quantity Ω_*ij*_(*t*) attributes an estimate of the fluctuation to each point of the spatiotemporal data set.

### Variants of the mutual information

The mutual information *I *([23]; see also [24]) has been shown to characterize the degree of complexity and information transport in complex biological systems [25,26]. The crucial idea is to analyze correlations over a spatial or temporal distance due to an interaction (or transfer of information) between the elements.

The general form of the mutual information *I *compares pair probabilities *p*_*ab *_of observing the symbol combination (*ab*) in the output sequence of some stochastic model with the corresponding quantity *p*_*a*_*p*_*b *_of a reference model of independent symbols:

I=∑a,b∈Σpablog⁡pabpapb,
 MathType@MTEF@5@5@+=feaafiart1ev1aaatCvAUfKttLearuWrP9MDH5MBPbIqV92AaeXatLxBI9gBaebbnrfifHhDYfgasaacH8akY=wiFfYdH8Gipec8Eeeu0xXdbba9frFj0=OqFfea0dXdd9vqai=hGuQ8kuc9pgc9s8qqaq=dirpe0xb9q8qiLsFr0=vr0=vr0dc8meaabaqaciaacaGaaeqabaqabeGadaaakeaacqWGjbqscqGH9aqpdaaeqbqaaiabdchaWnaaBaaaleaacqWGHbqycqWGIbGyaeqaaaqaaiabdggaHjabcYcaSiabdkgaIjabgIGiolabfo6atbqab0GaeyyeIuoakiGbcYgaSjabc+gaVjabcEgaNnaalaaabaGaemiCaa3aaSbaaSqaaiabdggaHjabdkgaIbqabaaakeaacqWGWbaCdaWgaaWcbaGaemyyaegabeaakiabdchaWnaaBaaaleaacqWGIbGyaeqaaaaakiabcYcaSaaa@4AB7@

where *p*_*a *_is the probability of observing the symbol *a*. The quantities *a *and *b *in Eq. (5) run over the whole set Σ of possible symbol states. When maximum likelihood estimates are used for the probabilities p_*a *_and *p*_*ab*_, it is convenient to project the data under consideration onto a binary state space Σ = {0, 1}.

Variants of applying the mutual information to the analysis of some spatiotemporal pattern differ in the precise definition of the joint event (*ab*), which leads to the pair probability *p*_*ab*_, and in the specific projection of the spatiotemporal pattern onto a binary state space.

**Figure 1:**
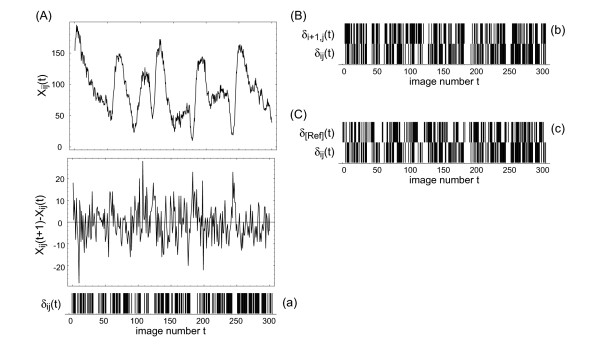
Binarization of the spatiotemporal data. The aim is to achieve a format suitable for computing the mutual information. (A) shows the time course *x*_*ij *_(*t*) of a single spatial point, together with the corresponding time series of temporal differences *x*_*ij*_(*t *+ 1) - *x*_*ij*_(*t*) for 300 images from the early-stage patterns of *D. discoideum*. The temporal differences are inserted into a binarization filter *δ*_*ij*_(*t*) = *Sig*(*x*_*ij*_(*t *+ 1) - *x*_*ij*_(*t*)). If *δ*_*ij*_(*t*) is zero, the value at the previous time step is used instead. For convenience one then passes to values 1 and 0, rather than 1 and -1. The lower row in (A) shows the resulting binarizations as bar code diagram. As described in the main text, variant (a) of the mutual information can be computed from this sequence of zeroes and ones. For variant (b) the binary patterns of two adjacent spatial points are considered (B). Variant (c) compares each spatial point with an arbitrarily selected reference point (C).

Figure 1 illustrates three different ways of defining the event (*ab*) entering the definition (5) using experimental data of *D. discoideum *pattern formation. In variant (a) the temporal neighborhood is used, i.e. two incidents *a *and *b *form a joint event (*ab*), when they are observed at two consecutive time points. Note that this variant does not involve spatial neighborhoods. In contrast, in variant (b) the event (*ab*) is defined via spatial neighborhood in *x*-direction. Variant (c) uses an arbitrarily chosen spatial reference point to define the joint event (*ab*). Here we focus on variant (a). It should be noted, however, that in very noisy data the variants (b) and (c) may show a better performance in extracting variability. As in all these cases the mutual information is computed for the time courses of individual spatial points the result of analyzing a full spatiotemporal data set is a spatial matrix *I*_*ij*_

## A simple model of an excitable medium

In order to study the link between intrinsic cellular properties and the local signature of these properties in the spatiotemporal patterns it is convenient to consider a simple model of an excitable medium given by a cellular automaton. In cellular automata the spatial discretization coincides with the discrete nature of cells and the observed states within the spatiotemporal pattern are reduced to few essential elements. In its simplest form, an excitable medium is a spatial arrangement of identical elements, for which (at least) three states exist, namely "quiescent" (excitable) (*Q*), "excited" (*E*) and "refractory" (*R*). A typical time sequence of states for a single cell is characterized by a switch from the quiescent state *Q *to the excited state *E *when a certain condition is fulfilled; the falling into the refractory state *R *after one time step and the remaining in *R *for a fixed period of time (called the refractory time). This sequence immediately leads to the formation of propagating wave fronts and, when disturbed, to spiral waves. In a more formal way, the three rules thus read as follows: (1) An element *a*_*ij *_in the state *Q *changes to *E*, when *E *appears in its neighborhood N
 MathType@MTEF@5@5@+=feaafiart1ev1aaatCvAUfKttLearuWrP9MDH5MBPbIqV92AaeXatLxBI9gBamrtHrhAL1wy0L2yHvtyaeHbnfgDOvwBHrxAJfwnaebbnrfifHhDYfgasaacH8akY=wiFfYdH8Gipec8Eeeu0xXdbba9frFj0=OqFfea0dXdd9vqai=hGuQ8kuc9pgc9s8qqaq=dirpe0xb9q8qiLsFr0=vr0=vr0dc8meaabaqaciaacaGaaeqabaWaaeGaeaaakeaaimaacqWFneVtaaa@383B@(*a*_*ij*_). (2) An element in the state *E *always proceeds to the state *R *in the next time step. (3) When *a*_*ij *_= *R, *the element will be in the state *Q *in the next time step, if the element has been in the state *R *for a time Δ*t*(*R*) equal to or larger than the refractory time *τ*.

The letters *Q*, *E *and *R *introduced above can now be used almost in a telegram style to describe the dynamics of the slime mold *D. discoideum*, where one has an ensemble of cells aggregating under the influence of a chemotactic cAMP signal that each cell is capable of producing and detecting: Sensing cAMP (which is, e.g., produced by one of its neighbors) the amoeba changes from *Q *to *E *(i.e., it emits cAMP itself), enters a rest phase *R *and starts to move perpendicular to the wave front. After a short period (of the order of minutes) the amoeba is sensitive to cAMP again, i.e., it again enters the stage *Q*. The existence of a refractory period, together with the interaction with neighbors (detection, emission and degradation of the cAMP signal) results in the formation of characteristic excitable-media patterns, namely concentric rings and spiral waves [27].

From the different cellular automaton models of excitable media (see, e.g., [28,29]) we select a variant, which combines simple update rules involving few parameters with a quasi-continuous state space helpful in achieving similarity with experimental data [30]. In this model we implement a simple interaction rule for epidemic spreading of excitations from cell to cell, which allows to study the global dynamics on a quasi-continuous state space (which in an epidemic scenario would correspond to the immunization state of the elements). The state space has the form

Σ = {0, 1,..., *n *- 1, *n*},

where healthy elements are represented by 0 (corresponding to the *Q *state of our general model introduced above), infected cells can be in states 1, ..., *n *- 1 (the *E *state), and the diseased (sick) elements are given by *n *(the *R *state).

**Figure 2:**
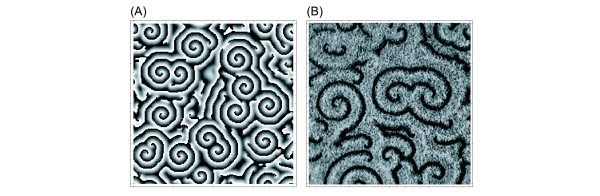
Snapshot of a pattern generated by the cellular automaton described in Section 3 after 2000 time steps (A) and of excitation waves in *D. discoideum *(B). In (A) the excitability was set to *g *= 28, the other parameter values are *k*_1 _= *k*_2 _= 2 and *n *= 100. The array size is 200 × 200 cells. Elements in the excitable state (0) are displayed in black, refractory elements (*n*) are white. The gray values correspond to the excited state (1, ..., *n *- 1). The section in (B) corresponds to 7.4 × 7.4 mm. The black wave fronts are cells, which have just detected the cAMP molecules. In the bright area the cells are moving chemotactically towards the respective spiral center.

The update rules which determine the state of a cell in the next time step are as follows:

xij=0→[ak1]+[bk2],xij=1→[sa+b+1]+g,i≠0,n,xij=n→0,
 MathType@MTEF@5@5@+=feaafiart1ev1aaatCvAUfKttLearuWrP9MDH5MBPbIqV92AaeXatLxBI9gBaebbnrfifHhDYfgasaacH8akY=wiFfYdH8Gipec8Eeeu0xXdbba9frFj0=OqFfea0dXdd9vqai=hGuQ8kuc9pgc9s8qqaq=dirpe0xb9q8qiLsFr0=vr0=vr0dc8meaabaqaciaacaGaaeqabaqabeGadaaakeaafaqabeqaeaaaaeaacqWG4baEdaWgaaWcbaGaemyAaKMaemOAaOgabeaakiabg2da9iabicdaWiabgkziUoaadmaabaWaaSaaaeaacqWGHbqyaeaacqWGRbWAdaWgaaWcbaGaeGymaedabeaaaaaakiaawUfacaGLDbaacqGHRaWkdaWadaqaamaalaaabaGaemOyaigabaGaem4AaS2aaSbaaSqaaiabikdaYaqabaaaaaGccaGLBbGaayzxaaGaeiilaWcabaGaemiEaG3aaSbaaSqaaiabdMgaPjabdQgaQbqabaGccqGH9aqpcqaIXaqmcqGHsgIRdaWadaqaamaalaaabaGaem4CamhabaGaemyyaeMaey4kaSIaemOyaiMaey4kaSIaeGymaedaaaGaay5waiaaw2faaiabgUcaRiabdEgaNjabcYcaSaqaaiabdMgaPjabgcMi5kabicdaWiabcYcaSiabd6gaUjabcYcaSaqaaiabdIha4naaBaaaleaacqWGPbqAcqWGQbGAaeqaaOGaeyypa0JaemOBa4MaeyOKH4QaeGimaaJaeiilaWcaaaaa@6851@

where [*x*] is the integer remainder of *x *(i.e. the remaining integer value after removing the decimal part), a denotes the number of infected cells in the neighborhood Nij
 MathType@MTEF@5@5@+=feaafiart1ev1aaatCvAUfKttLearuWrP9MDH5MBPbIqV92AaeXatLxBI9gBaebbnrfifHhDYfgasaacH8akY=wiFfYdH8Gipec8Eeeu0xXdbba9frFj0=OqFfea0dXdd9vqai=hGuQ8kuc9pgc9s8qqaq=dirpe0xb9q8qiLsFr0=vr0=vr0dc8meaabaqaciaacaGaaeqabaqabeGadaaakeaat0uy0HwzTfgDPnwy1egaryqtHrhAL1wy0L2yHvdaiqaacqWFneVtdaWgaaWcbaGaemyAaKMaemOAaOgabeaaaaa@3B1E@ of a point (*ij*) and *b *represents the corresponding number of sick cells in Nij
 MathType@MTEF@5@5@+=feaafiart1ev1aaatCvAUfKttLearuWrP9MDH5MBPbIqV92AaeXatLxBI9gBaebbnrfifHhDYfgasaacH8akY=wiFfYdH8Gipec8Eeeu0xXdbba9frFj0=OqFfea0dXdd9vqai=hGuQ8kuc9pgc9s8qqaq=dirpe0xb9q8qiLsFr0=vr0=vr0dc8meaabaqaciaacaGaaeqabaqabeGadaaakeaat0uy0HwzTfgDPnwy1egaryqtHrhAL1wy0L2yHvdaiqaacqWFneVtdaWgaaWcbaGaemyAaKMaemOAaOgabeaaaaa@3B1E@. The quantity *s *in the update rule for infected cells is the sum over all elements in Nij
 MathType@MTEF@5@5@+=feaafiart1ev1aaatCvAUfKttLearuWrP9MDH5MBPbIqV92AaeXatLxBI9gBaebbnrfifHhDYfgasaacH8akY=wiFfYdH8Gipec8Eeeu0xXdbba9frFj0=OqFfea0dXdd9vqai=hGuQ8kuc9pgc9s8qqaq=dirpe0xb9q8qiLsFr0=vr0=vr0dc8meaabaqaciaacaGaaeqabaqabeGadaaakeaat0uy0HwzTfgDPnwy1egaryqtHrhAL1wy0L2yHvdaiqaacqWFneVtdaWgaaWcbaGaemyAaKMaemOAaOgabeaaaaa@3B1E@. The remaining quantities *k*_1_, *k*_2 _and *g *are model parameters, which regulate the impact of infected and sick cells on neighbors and the excitability of a cell, respectively. In a certain range of the parameter space the model exhibits a dynamics, which is highly comparable to that of real excitable media, particularly early patterns in *D. discoideum *or the spatially extended Belousov-Zhabotinsky (BZ) reaction (cf. Figure 2).

We introduce variability in this system as distributions of *g *and of *k*_2_. In both cases we use two different integer values (a high value g* and k2∗
 MathType@MTEF@5@5@+=feaafiart1ev1aaatCvAUfKttLearuWrP9MDH5MBPbIqV92AaeXatLxBI9gBaebbnrfifHhDYfgasaacH8akY=wiFfYdH8Gipec8Eeeu0xXdbba9frFj0=OqFfea0dXdd9vqai=hGuQ8kuc9pgc9s8qqaq=dirpe0xb9q8qiLsFr0=vr0=vr0dc8meaabaqaciaacaGaaeqabaqabeGadaaakeaacqWGRbWAdaqhaaWcbaGaeGOmaidabaGaey4fIOcaaaaa@3019@, respectively, and a low background value *g*^*B *^and k2B
 MathType@MTEF@5@5@+=feaafiart1ev1aaatCvAUfKttLearuWrP9MDH5MBPbIqV92AaeXatLxBI9gBaebbnrfifHhDYfgasaacH8akY=wiFfYdH8Gipec8Eeeu0xXdbba9frFj0=OqFfea0dXdd9vqai=hGuQ8kuc9pgc9s8qqaq=dirpe0xb9q8qiLsFr0=vr0=vr0dc8meaabaqaciaacaGaaeqabaqabeGadaaakeaacqWGRbWAdaqhaaWcbaGaeGOmaidabaGaemOqaieaaaaa@3037@, respectively). A certain percentage of spatial sites is assigned the high value. The percentage of high-value sites constitutes the strength of variability, while the spatial distribution of parameter values is the matrix we aim at reconstructing with the help of our spatiotemporal observables defined in the previous sections. The spatial matrices *K*_*ij *_and *G*_*ij *_for *k*_2 _and *g *distributions, respectively, constitute our model implementation of cell-cell differences (in two different cellular properties). In the following, *ν*_*k *_and *ν*_*g *_denote the respective percentage of k2∗
 MathType@MTEF@5@5@+=feaafiart1ev1aaatCvAUfKttLearuWrP9MDH5MBPbIqV92AaeXatLxBI9gBaebbnrfifHhDYfgasaacH8akY=wiFfYdH8Gipec8Eeeu0xXdbba9frFj0=OqFfea0dXdd9vqai=hGuQ8kuc9pgc9s8qqaq=dirpe0xb9q8qiLsFr0=vr0=vr0dc8meaabaqaciaacaGaaeqabaqabeGadaaakeaacqWGRbWAdaqhaaWcbaGaeGOmaidabaGaey4fIOcaaaaa@3019@ and g* values in the parameter matrices. These quantities allow tuning the strength of variability.

**Figure 3:**
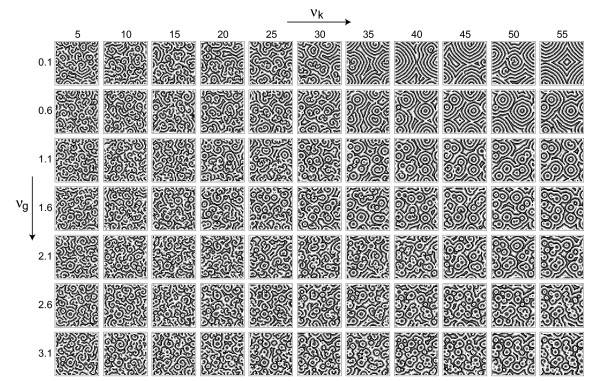
Cellular automaton model of an excitable medium for different variablities. Snapshots of a 132 × 132 lattice after 1000 time steps are shown with *n *= 100, *k*_1 _= 2, *g*^*B *^= 28, k2B
 MathType@MTEF@5@5@+=feaafiart1ev1aaatCvAUfKttLearuWrP9MDH5MBPbIqV92AaeXatLxBI9gBaebbnrfifHhDYfgasaacH8akY=wiFfYdH8Gipec8Eeeu0xXdbba9frFj0=OqFfea0dXdd9vqai=hGuQ8kuc9pgc9s8qqaq=dirpe0xb9q8qiLsFr0=vr0=vr0dc8meaabaqaciaacaGaaeqabaqabeGadaaakeaacqWGRbWAdaqhaaWcbaGaeGOmaidabaGaemOqaieaaaaa@3037@ = 2, g* = 40 and k2∗
 MathType@MTEF@5@5@+=feaafiart1ev1aaatCvAUfKttLearuWrP9MDH5MBPbIqV92AaeXatLxBI9gBaebbnrfifHhDYfgasaacH8akY=wiFfYdH8Gipec8Eeeu0xXdbba9frFj0=OqFfea0dXdd9vqai=hGuQ8kuc9pgc9s8qqaq=dirpe0xb9q8qiLsFr0=vr0=vr0dc8meaabaqaciaacaGaaeqabaqabeGadaaakeaacqWGRbWAdaqhaaWcbaGaeGOmaidabaGaey4fIOcaaaaa@3019@ = 3. The percentage of high-value elements for *g *has been varied between 0.1 and 3.1 percent in steps of 0.5 percent (top to bottom), while the percentage of high-value elements for *k*_2 _has been varied between 5 and 55 percent in steps of 5 percent (left to right).

Indeed, the patterns produced by the model depend systematically on the two sources of variability. Figure 3 shows typical snapshots of simulations for different strengths of variability.

At low *ν*_*g *_the effect of *ν*_*k *_is very pronounced: with increasing *ν*_*k *_one has larger domains and a bias towards target waves (compared to the spiral-dominated patterns at low *ν*_*k*_). At higher values of *ν*_*g *_the effect of *ν*_*k *_on domain size is less pronounced, but the bias towards target waves remains. Note that our scheme of implementing variability allows us to pass to a higher variablity strength simply by inserting further high-value sites into the previous grid. When passing from one value of variability to a higher value of variability we keep the previous pacemaker positions fixed and add the correct amount of new pacemakers randomly. Qualitatively speaking, we implement variability here as a pacemaker density, e.g., as the density of highly excitable cells. Note, however, that in our setup such pacemakers can differ from the other cells in two properties: *g *(corresponding to the excitability) and *k*_2 _(corresponding to a sensitivity). These two contributions to the overall excitability are well known in the case of *D. discoideum *[31]. The matrices *G*_*ij *_and *K*_*ij *_store the positions of these pacemaker elements. The percentages of high-value entries (k2∗
 MathType@MTEF@5@5@+=feaafiart1ev1aaatCvAUfKttLearuWrP9MDH5MBPbIqV92AaeXatLxBI9gBaebbnrfifHhDYfgasaacH8akY=wiFfYdH8Gipec8Eeeu0xXdbba9frFj0=OqFfea0dXdd9vqai=hGuQ8kuc9pgc9s8qqaq=dirpe0xb9q8qiLsFr0=vr0=vr0dc8meaabaqaciaacaGaaeqabaqabeGadaaakeaacqWGRbWAdaqhaaWcbaGaeGOmaidabaGaey4fIOcaaaaa@3019@ and g*) in these matrices correspond to the variability strengths varied in Figure 3 and discussed quantitatively in the following analyses.

In this way we can understand more clearly, how the reconstruction of cell-cell differences, e.g., depends on the number of high-excitability cells in the system (rather than a new arrangement of these elements).

## Experimental methods

*D. discoideum *cells of the axenic strain AX2 were cultivated from frozen spores (-18°C) according to the standard procedure [32] in HL5-medium at 21°C to a final cell density of 6·10^6^ cells/ml. To initiate starvation and therefore pattern formation the cells have been harvested by low speed centrifugation and washed twice with phosphate buffer (15.7 mM *KH*_2_*PO*_4_/*Na*_2_*HPO*_4_, pH 6.14). The resuspended cells were spread homogeneously onto water agar plates (1% Difco Bacto agar, 2 mM caffeine in phophate buffer, pH 6.14) to a density of 6.2·10^5 ^cells/cm^2^. The supernatant was removed and the plates were incubated in darkness. After five to six hours of starvation cAMP waves become indirectly visible in dark-field [33,34]. Individual cells change their light scattering properties in dependence of the local excitation state, resulting in macroscopic wave pattern. Our dark-field optics was constructed according to [35]. Successive images were taken in equidistant time intervals of 3 sec (Hamamatsu C 3077, DT-Open Layers DT 3155 Mach Series Frame Grabber).

## Results

### Results for the model simulation

From Figure 3 we see that the two forms of variability described in Section 3 have a systematic influence on properties of the resulting patterns. Note that here we are not interested in studying the effect of variability on the patterns in detail. In such a case the mean value of each parameter should be kept constant. The interesting property for our present aim of reconstructing cell-cell differences is the mere presence of a parameter distribution. With the more general framework in mind, it is of course noteworthy that (for this deterministic system) the parameter distribution *determines *the exact layout of the patterns. We believe that by virtue of the process of self-organization even in a non-deterministic (stochastic) system this correspondence can exist and inscribes a certain amount of predictability into the system.

**Figure 4:**
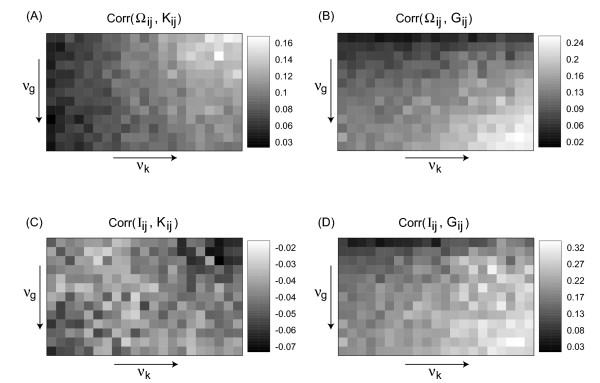
Correlation coefficients between the matrices *Obs*_*ij *_(Ω_*ij *_and *I*_*ij*_, respectively) and *Par*_*ij *_(*K*_*ij *_and *G*_*ij*_, respectively) for the array of patterns shown in Figure 3. Here, *ν*_*g *_and *ν*_*k *_have been varied in the same ranges as in Figure 3, but with half the step size.

In order to see, how well the spatiotemporal observables from Section 2 reconstruct the parameter distributions we compute the correlation coefficient between the matrices Obs_*ij *_and Par_*ij*_, where Obs_*ij *_is either Ω_*ij *_or *I*_*ij *_and Par_*ij *_can be *G*_*ij *_or K_*ij*_, as introduced in Section 3. For the array of data from Figure 3 we thus obtain the four correlation arrays given in Figure 4, which are highly systematic: The correlation between Ω_*ij *_and *K*_*ij *_remains essentially constant with *ν*_*g*_, as it should be, but changes systematically with *ν*_*k*_. The opposite is true for the correlation between Ω*_ij_* and *G*_*ij *_. The other observable, the mutual information *I*_*ij*_, reacts strongest to *G*_*ij*_, but also the comparison with *K*_*ij *_shows a small but consistently negative correlation coefficient varying systematically within the array. In both cases the observed correlation coefficients can be quite high (up to 0.2 or 0.3) showing that our attempt of reconstructing the variability distributions with these spatiotemporal observables is rather successful.

**Figure 5:**
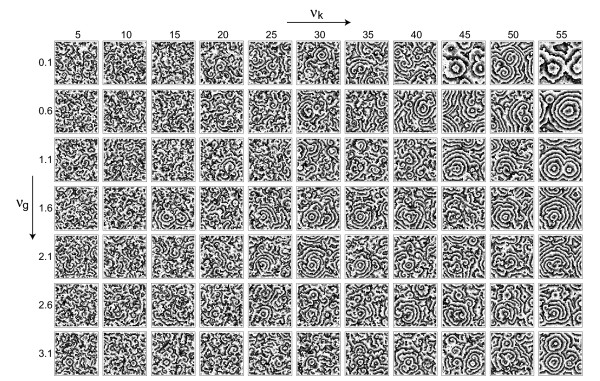
Same as Figure 3, but with the influence of noise, as described in the main text. Parameter values are the same as in Figure 3.

An important issue for practical applications of these tools is their robustness with respect to noise. In our model, noise can be implemented, e.g., by adding a random integer number to each *x*_*ij *_in each time step with the subsidiary condition of remaining in the model's state space. Here we are using *n *= 100, as before, and the random integers added as noise are equally distributed between -10 and 10. Figure 5 shows an array of typical spatial snapshots under the influence of this noise.

The general features of the patterns remain the same as in Figure 3. This is important for our analysis, as the noise does not destroy the patterns completely. Note also that the general trends of variability effects remain similar to Figure 3. Details of the wave propagation and spiral formation, however, are strongly affected by the noise. Surprisingly, the reconstruction of the underlying variability distributions is not impeded by noise. One rather observes in many cases an enhancement of the reconstruction. This is particularly true for the fluctuation number Ω_*ij*_, which needs a certain amount of noise to properly detect systematic differences between spatial neighbors. Noise in this case helps sample the possibility space of neighbor differences entering the filter defined in equation 4. The mutual information does not show such an enhancement, but is still capable of filtering out the noise contribution. It is also clearly seen that reconstruction of the two sources of variability differs in their sensitivity to noise: While excitability (*G*_*ij*_) is generally well reconstructed in the noisy case, for the sensitivity (*K*_*ij*_) we observe no enhancement compared to the noise-free case.

**Figure 6:**
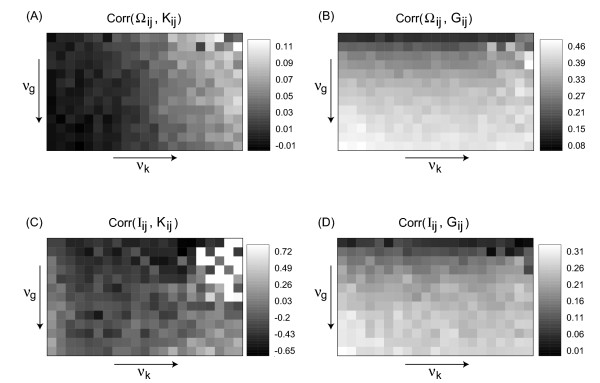
Same as Figure 4, but for the spatiotemporal patterns under the influence of noise, as shown in Figure 5.

**Figure 7:**
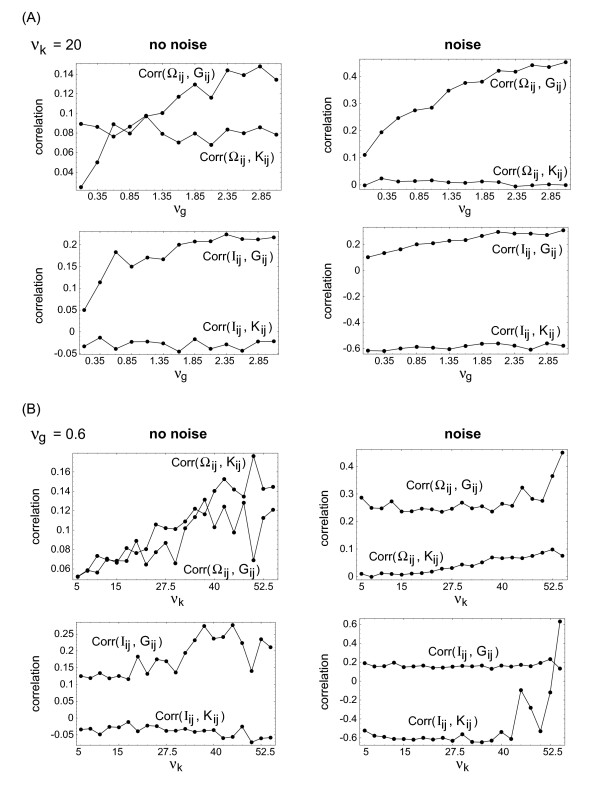
Correlation coefficients between the reconstructed matrices *Obs*_*ij *_and the parameter distributions *Par*_*ij *_as a function of the two variability strengths *ν*_*g *_(at fixed *ν*_*k *_= 20) and *ν*_*k *_(at fixed *ν*_*g *_= 0.6), both for the patterns without noise (left-hand side) and with noise (right-hand side). The curves are cross sections of the correlation arrays from Figures 4 and 6.

Figure 6 shows the corresponding arrays of correlation coefficients. This enhancement due to noise (which is reflected in higher correlation coefficients and a more systematic parameter dependence) can be seen more clearly, when the correlation coefficients are plotted as a function of one variability strength, while keeping the other constant (i.e. by looking at cross sections of the correlation arrays from Figures 4 and 6). This is summarized in Figure 7.

Even in our minimal system (two sources of variability, two observables) we already see that the observables may depend differently on different sources of variability. In real-life systems such observations may help setting up a combinatorical scheme for estimating, which source of variability contains the strongest signal for predicting later-stage patterns.

**Figure 8:**
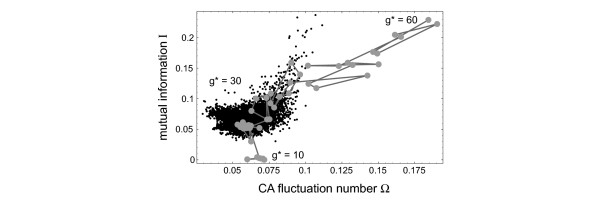
Reconstruction of a single pacemaker element in the (Ω, *I*) plane as a function of its pacemaker strength g*, which was changed between g* = 60 and 10. The excitability of the background elements was *g*^*B *^= 25. All other parameters are the same as in Figures 2 and 3 (at k2∗
 MathType@MTEF@5@5@+=feaafiart1ev1aaatCvAUfKttLearuWrP9MDH5MBPbIqV92AaeXatLxBI9gBaebbnrfifHhDYfgasaacH8akY=wiFfYdH8Gipec8Eeeu0xXdbba9frFj0=OqFfea0dXdd9vqai=hGuQ8kuc9pgc9s8qqaq=dirpe0xb9q8qiLsFr0=vr0=vr0dc8meaabaqaciaacaGaaeqabaqabeGadaaakeaacqWGRbWAdaqhaaWcbaGaeGOmaidabaGaey4fIOcaaaaa@3019@ = k2B
 MathType@MTEF@5@5@+=feaafiart1ev1aaatCvAUfKttLearuWrP9MDH5MBPbIqV92AaeXatLxBI9gBaebbnrfifHhDYfgasaacH8akY=wiFfYdH8Gipec8Eeeu0xXdbba9frFj0=OqFfea0dXdd9vqai=hGuQ8kuc9pgc9s8qqaq=dirpe0xb9q8qiLsFr0=vr0=vr0dc8meaabaqaciaacaGaaeqabaqabeGadaaakeaacqWGRbWAdaqhaaWcbaGaeGOmaidabaGaemOqaieaaaaa@3037@ = 2).

An interesting numerical experiment is to introduce a single highly excitable element in the system and then follow the reconstruction of this element in the (Ω, *I*) plane. In this experiment all *K*_*ij *_are set to k2B
 MathType@MTEF@5@5@+=feaafiart1ev1aaatCvAUfKttLearuWrP9MDH5MBPbIqV92AaeXatLxBI9gBaebbnrfifHhDYfgasaacH8akY=wiFfYdH8Gipec8Eeeu0xXdbba9frFj0=OqFfea0dXdd9vqai=hGuQ8kuc9pgc9s8qqaq=dirpe0xb9q8qiLsFr0=vr0=vr0dc8meaabaqaciaacaGaaeqabaqabeGadaaakeaacqWGRbWAdaqhaaWcbaGaeGOmaidabaGaemOqaieaaaaa@3037@ = 2 and all but one *G*_*ij *_to *g*^*B *^= 25, while a single position is assigned a different value g*, which is changed between g* = 60 and g* = 10. Figure 8 displays the corresponding reconstruction from simulated data in the (Ω, *I*) plane. The cloud of small black dots denotes the (Ω, *I*) values for all other (non-pacemaker) elements. Starting now from a very high value of excitability g* = 60 for the single pacemaker element we gradually reduce the value of g* and observe the corresponding trajectory in the (Ω, *I*) plane. Over a wide range of g* (both at high and low values) this single pacemaker element stands out in terms of these reconstructed values. Only when g* is close to the values of the background elements, the pacemaker is within the cloud formed by the other elements.

### Application to *D. discoideum *pattern formation

**Figure 9:**
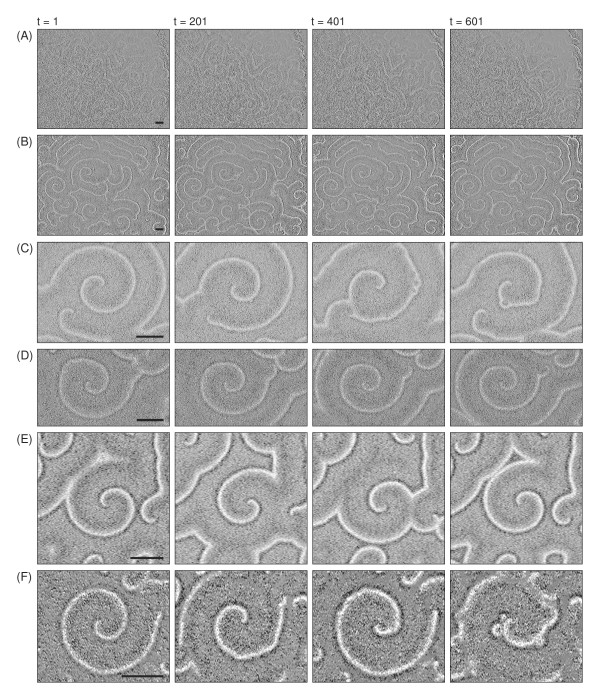
Snapshots of experimental data sets analyzed on their spatial distribution in cell-cell differences (bar size 2 mm). Time points are indicated above the array of snapshots. In addition the spatial size the experimental data differ in their resolution: (A) and (B) 22.6 pixels/mm, (C) 68.2 pixels/mm, (D) 68.0 pixels/mm, (E) 38.6 pixels/mm, (F) 53.3 pixels/mm. As a rule of thumb at a cell density of 6.172·10^5 ^cells/*cm*^2 ^one can expect that 1 pixel contains approximately 12 cells in (A) and (B), 1 cell in (C) and (D), 4 cells in (E) and 2 cells in (F).

**Figure 10:**
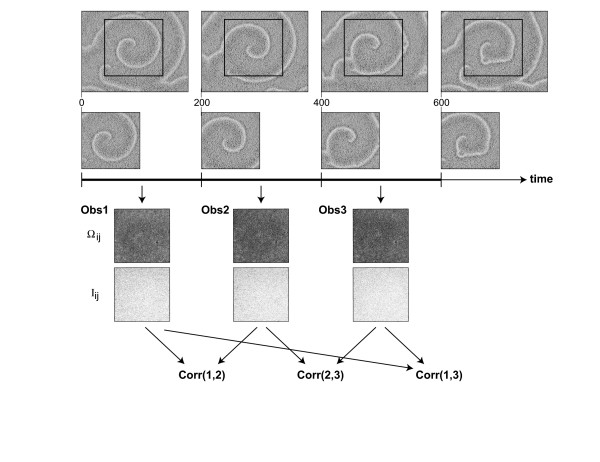
Schematic view of the procedure of individual cell property extraction. Data sets were devided into intervals of 200 images corresponding to 10 minutes in the experiments considered here. For each interval the observables Ω_*ij *_and *I*_*ij *_were determined. For this particular data set (denoted (C) in Figure 9) the corresponding observables (for a particular image segment) are shown below the time axis: first row – Ω_*ij*_, second row – *I*_*ij *_. One sees from these distributions that the two observables focus on small-scale fluctuations rather than the large-scale features of the original patterns. In the subsequent analysis the three correlation coefficients of the reconstructed matrices (first and second time interval, second and third, and first and third) are computed both for Ω_*ij *_and *I*_*ij*_.

**Figure 11:**
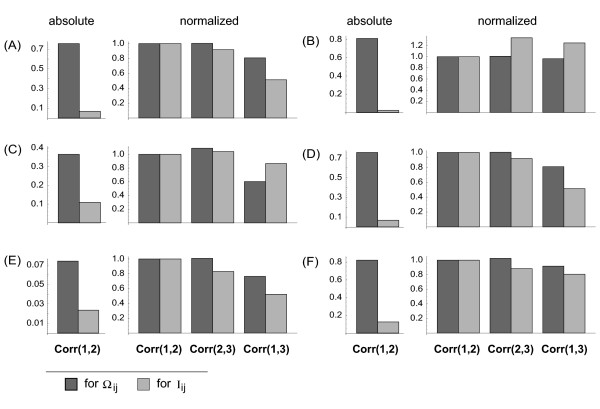
Correlation coefficients of Ω_*ij *_and *I*_*ij*_, respectively, between the different intervals of experimental data. On the left-hand side of each image segment one can see the absolute values of correlation coefficients between intervals one and two (Corr(1,2), cf. Figure 10). The columns on the right-hand image parts show correlation coefficients between the intervals normalized to Corr(1,2). The notation (A) to (F) corresponds to that of Figure 9.

The patterns of an early stage of the *D. discoideum *life cycle, where cell-cell communication leads to propagating waves, are an ideal field of application for the reconstruction methods of cell-cell variability described in the previous sections. As the dark-field images do not allow observing individual cells directly, we apply the analysis tools to each pixel and assume that the observables provide estimates valied as avarage for the cells residing at this spot. Cell movement is neglected in this analysis. This is not unrealistic, as at this stage of pattern formation directed movement is small. Figure 9 shows snapshots of corresponding experimental data sets. In order to see, if the observables from Section 2 indeed yield reproducible results, even though they essentially evaluate the systematics of neighborhood fluctuations behind the overall self-organized dynamics in a spatiotemporal data set, we compute Ω_*ij *_and *I*_*ij *_for three different time intervals and then see, if the reconstructed matrices correlate over time. Figure 10 summarizes the general scheme. Inspite of their respective focus on small-scale fluctuations (cf. the definitions of *I*_*ij *_and Ω_*ij *_in Section 2) the two observables show a very systematic result, which suggests that the individual pixels possess a specific dynamic response, even though the system as a whole displays a self-organized pattern with a high spatial order on a larger scale (Figure 11): For consecutive intervals (1, 2) and (2, 3) the correlation coefficients are almost identical, while they are (in most cases) systematically reduced for a larger time difference (1, 3). The result from Figure 11 complements nicely the single-cell observations from [16]. While these authors look at individual *D. discoideum *cells under well-defined stimuli, we analyze statistically a very large ensemble of cells in the process of pattern formation. In this way, our result is a cell-population variant of the findings in [16]. It is surprising that the individual cell properties contribute strongly and systematically enough to show up in this analysis.

## Conclusion and outlook

The aim of the paper is two-fold: First, we want to introduce the general idea that spatial distributions of cellular properties may serve as a basis for predicting later stages of a pattern formation process; second, we provide some tools for reconstructing such distributions from spatiotemporal data sets. The following results have been shown: The fluctuation number and the mutual information are adequate tools for extracting spatial variations in cell properties from experimental data; different cell properties are picked up differently by these tools (a point we discuss in detail for excitability and sensitivity); reconstruction of such cell properties by the fluctuation number is under certain conditions enhanced by noise; applying these tools to experimental data of *D. discoideum *pattern formation we find that, in agreement with the single-cell results from [16], individual loci in the spatial pattern possess systematic properties, which contribute to the pattern formation process.

While we are, at this stage of the investigation, far from explicitly formulating such a prediction scheme and applying it to specific biological scenarii, we nevertheless believe that this new view on pattern formation is of relevance to many biological systems. We do not imply that the techniques lined out here are the only means of reconstructing variability distributions from data. In fact, the choice of tools will strongly depend on the specific system at hand. Criteria for selecting the analysis tools could be: Is noise an important factor in the data? Which forms of variability are expected to be relevant? Which is the dominant form of dynamics; which types of patterns are formed?

For generic models of excitable media and, to a certain extent, for experimental data on *D. discoideum *pattern formation we have shown that simple spatiotemporal observables can be employed to reconstruct the spatial distribution of biological properties involved the pattern formation process. How does one pass from these methodological findings to a practical application of the prediction scheme lined out in the introduction? As long as there is no general theory connecting such distribution with the layout with later-stage patterns one has to rely on heuristic protocols. It should be possible for some systems where a large enough number of data sets is available to train an artificial neural network or a system of self-organizing maps to the task of linking distribution patterns with properties of the later-stage self-organized state. Which features of self-organized patterns can in principle be thought of as predictees of early-stage distributions in cellular properties? When one focuses on excitable media clearly the specific locations of phase singularities (i.e. the terminal points of spiral wave fronts) are a candidate for such predictive schemes. Particularly because recent work on *D. discoideum *relates the density of such phase singularities with the strength of a genetic feedback loop at the single cell level [31], it would be of huge interest to understand how a distribution of cell properties in this specific scenario translates into a distribution of phase singularities.

The new view on biological pattern formation as a consequence, beyond the general rules of the process, of distributed properties goes along with the potential of predicting features of the later-stage patterns. It should be noted, however, that such predictions will always be of statistical nature and never one-to-one correspondences. Nevertheless, for the range of biological systems, for which such predictions could be of interest (ranging from excitation waves in heart tissue to calcium waves on cellular membranes and waves of epidemic spreading of diseases), even the possibility of assigning e.g. the probability of a phase singularity to a particular position has interesting implications for intervention schemes and risk assessment.
